# Programmed Degradation of Pericarp Cells in Wheat Grains Depends on Autophagy

**DOI:** 10.3389/fgene.2021.784545

**Published:** 2021-12-13

**Authors:** Yong-Bo Li, Mei Yan, De-Zhou Cui, Chen Huang, Xin-Xia Sui, Feng Zhi Guo, Qing-Qi Fan, Xiu-Sheng Chu

**Affiliations:** ^1^ Crop Research Institute, Shandong Academy of Agricultural Sciences, Jinan, China; ^2^ Shandong Luyan Seed Company, Jinan, China; ^3^ Heze Academy of Agricultural Sciences, Heze, China; ^4^ School of Life Science, Shandong Normal University, Jinan, China

**Keywords:** wheat, pericarp, autophagy, programmed cell death, autophagy-related genes

## Abstract

Wheat is one of the most important food crops in the world, with development of the grains directly determining yield and quality. Understanding grain development and the underlying regulatory mechanisms is therefore essential in improving the yield and quality of wheat. In this study, the developmental characteristics of the pericarp was examined in developing wheat grains of the new variety Jimai 70. As a result, pericarp thickness was found to be thinnest in grains at the top of the spike, followed by those in the middle and thickest at the bottom. Moreover, this difference corresponded to the number of cell layers in the pericarp, which decreased as a result of programmed cell death (PCD). A number of autophagy-related genes (*ATGs*) are involved in the process of PCD in the pericarp, and in this study, an increase in ATG8-PE expression was observed followed by the appearance of autophagy structures. Meanwhile, following interference of the key autophagy gene *ATG8*, PCD was inhibited and the thickness of the pericarp increased, resulting in small premature grains. These findings suggest that autophagy and PCD coexist in the pericarp during early development of wheat grains, with both processes increasing from the bottom to the top of the spike. Moreover, PCD was also found to rely on *ATG8*-mediated autophagy. The results of this study therefore provide a theoretical basis for in-depth studies of the regulatory mechanisms of wheat grain development.

## Introduction

Wheat, one of the most important cereal crops worldwide, is characterized by its process of grain development. Although grains in the middle spikelet are first to bloom, the upper grains mature first, followed by the middle, and then the lower grains. The wheat grain is a type of caryopsis, whereby the pericarp and episperm develop from the integument and are tightly integrated (Zhou et al., 2009). Development of the pericarp is closely related to grain yield and overall wheat quality. Developing from the ovary wall, it can be divided into the exocarp, mesocarp and endocarp (Xiong et al., 2013). The pericarp covers the seed tegument, and endosperm and embryo tissues of the grain (Brinton et al., 2017) and controls the water transport into the endosperm cavity (Wang and Fisher, 1994), the synthesis of organic compounds (Fujita and Taira, 1998; Foxon et al., 1990), and the temporal storage of starch (Yu et al., 2015).

It was previously suggested that development of the pericarp is a typical process of programmed cell death (PCD) (Pennell and Lamb, 1997; Zhou et al., 2009), a genetically-regulated process of cell suicide that results in the remobilization of cellular contents, nourishing new filial tissues, such as the embryo and endosperm, and providing space for grain filling (Radchuk et al., 2011; Dominguez and Cejudo, 2014). Meanwhile, autophagy is responsible for the delivery of cellular components to the lysosome/vacuole for subsequent degradation, especially under nutrient limitations and other stress conditions, thereby supporting cellular proteostasis and longevity (Ghosh et al., 2015). Autophagic processes mainly serve survival functions during cellular homeostasis, stress adaptation and immune responses, but have also been found to possess cell death-promoting activities (Bozhkov, 2018). Genetic suppression of autophagy in plants is correlated with an overall decrease in plant fitness, including reduced vegetative growth and fecundity, accelerated senescence and enhanced susceptibility to diverse types of stress (Bozhkov, 2018). However, the role of autophagy in regulating PCD in plants remains unknown and a subject of debate (Ustun et al., 2017).

In wheat, autophagy is involved in the regulation of various biotic and abiotic stresses. Under hypoxia, wheat roots can remove reactive oxygen species by autophagy, thus maintaining cell survival (Lin et al., 2021). Under salt stress, interfering of autophagy-related genes *ATG2* or *ATG7* causes PCD in leaves (Yue et al., 2021). Inhibition of autophagy can accelerate PCD of seedlings caused by drought (Li et al., 2019). Short-term waterlogging and cold stress promote autophagy of wheat root cells (Valitova et al., 2019; Zhou et al., 2021). Autophagy-related genes *ATG4*, *ATG6*, and *ATG8* of wheat participate in the regulation of basic resistance to powdery mildew (Pei et al., 2014). *ATG8* contributes to wheat resistance to stripe rust fungus by regulating cell death (Ma et al., 2012). However, it is unclear whether autophagy is involved in the regulation of wheat grain development.

In this study, we used the new wheat variety Jimai 70 to examine the regulation of autophagy on grain development and PCD in pericarp. Our data suggested that autophagy and PCD coexist in the development of the pericarp; and both processes increasing from the bottom to the top of the spike, which determines the thickness of the pericarp at the corresponding position.

## Materials and Methods

### Plant Materials

Wheat cultivar Jimai 70 was developed by the Crop Research Institute, Shandong Academy of Agricultural Sciences, China. It possesses a number of elite traits, such as high and stable yield, lodging resistance, strong wind resistance, and slow stripe rust resistance.

### Quantitative Real-Time Reverse Transcription-PCR

Total RNA was extracted from the pericarp using RNAprep Pure Plant Kit (DP432, TIANGEN, Beijing, China) according to the manufacturer’s instructions. After determining RNA quality by electrophoresis on 1% agarose gel, 2 μg of RNA was reverse transcribed into cDNA using EasyScript One-Step gRNA Removal and cDNA Synthesis Super Mix (L20602, Transgen, Beijing, China). The resulting cDNA was then used as a template in the PCR reactions. qRT-PCR was performed using TransStart Tip Green qPCR SuperMix (L20803, Transgen) according to the manufacturer’s instructions in a real-time thermal cycler (LightCycler R 480 II, Roche, Basel, Switzerland). α-Tubulin was amplified for internal standardization. The experiments were repeated three times and the experimental data were statistically analyzed using the Student’s t-test. Relative expression data from the qRT-PCR experiments were obtained using the 2^−ΔΔCT^ method (Ustun et al., 2017). Relevant primers were listed in Table 1.

**TABLE 1:** **Primers used in this study.** *ATG* (*4*, *6*, *7*, *8*, *12*) QRT primers were used for quantitative real-time reverse transcription-PCR (qRT-PCR) of autophagy-related genes (*ATGs*). RNAi primers of *ATG8* were used to amplify the interference sequence of autophagy-related *ATG8*.

Primer name	Sequence (5′ - 3′)
ATG4QRTf	GAAAGCCCGCACAGAGTC
ATG4QRTr	ACC​CGA​GAC​CAC​ATA​GAG​C
ATG6QRTf	TTTCCGTCTCGGTCGTCT
ATG6QRTr	CAA​ACT​TAT​GGC​AAA​CTC​G
ATG7QRTf	TGCCTCACTGGTGCTTAG
ATG7QRTr	CAA​TCC​TTG​AGT​TGC​CTT​A
ATG8QRTf	AGG​CTG​ATA​AGT​CTG​ATG​TCC
ATG8QRTr	CGTCCTCGTCCTTGTTTT
ATG12QRTf	ACA​AGT​TCA​GGA​TTT​CAG​GAC​GAG
ATG12QRTr	TGC​CGA​CAA​AGC​ATA​GTT​TAC​CAC
ATG8RNAiF	ATG​GCG​AAG​AGC​TCG​TTC​AAG
ATG8RNAiR	TGG​CAG​ACA​TCA​GGG​CAG​C
DsGFPF	ATG​GTG​AGC​AAG​GGC​GAG​G
DsGFPR	GGA​CGT​AGC​CTT​CGG​GCA​TGG
ɑ-TubulinF	AACTTCGCCCGTGGTCAT
ɑ-TubulinR	CAG​CGT​TGA​ATA​CAA​GGA​ATC

### Preparation of Polyclonal Antibodies of ATG8 and α-Tubulin

Synthetic peptide (5 mg) samples obtained from the ATG8 protein sequence were first coupled to Keyhole Limpet Hemocyanin. The coupling polypeptide was then used as an antigen to produce rabbit polyclonal antibodies (anti-ATG8 antibodies) as described previously (Liu et al., 2014). A partial cDNA sequence containing 90 - 750 bp of α-tubulin was then amplified with the selected primers α-tubulin F/R (Table 1), and inserted into the *pGEX4T-AB1* plasmid. The recombinant plasmid was then transformed into competent *Escherichia coli* (BL21-DE3). The expressed α-tubulin was then obtained as a supernatant and purified before using the target protein as an antigen to produce rabbit polyclonal antibodies (anti-α-tubulin antibodies) as with the ATG8 antibodies.

### Western Blot (Immunoblotting)

Total proteins from the pericarp were extracted using Plant Total Protein Lysis Buffer (P1258, Applygen Technologies Inc., Beijing, China). The protein concentration was then measured according to the Bradford method (Bradford, 1976), and equal amounts (30 μg) of each sample were subjected to SDS-PAGE. Proteins were then electrophoretically transferred onto a nitrocellulose membrane and incubated with blocking buffer [2% skim milk powder dissolved in TBS (8.8 g NaCl, 5 ml of 2 M Tris-HCl, pH 7.6, and 995 ml of H_2_O)] at room temperature for 1 h. Rabbit source polyclonal antibody ATG8 or α-tubulin was then diluted to 1:500 in blocking buffer in TBS and incubated with the membrane at 4°C overnight. After washing, the membrane was incubated with secondary antibody (alkaline phosphatase conjugated goat anti-rabbit IgG diluted 1:10,000 in blocking buffer) (ZB2308, Zhong Shan Jin Qiao, Beijing, China) at room temperature for 2.5 h then the protein signal was visualized using an Alkaline Phosphatase Color Development Kit (C3206, Beyotime, Shanghai, China). Protein bands on the membrane were then analyzed using Image J software.

### Virus Induced Gene Silencing of *ATG8*

The barley stripe mosaic virus (BSMV)-based VIGS method was used to create gene knockdown plants (Ma et al., 2012; Dong et al., 2019). Briefly, a 283-bp fragment of wheat *ATG8* from the conserved coding sequence was amplified and purified, with a same-sized fragment of GFP used as a control. The γ strand of BSMV was then digested in XmacI and fused with the *ATG8* or *GFP* fragment to form the vectors BSMVγ-ATG8 and BSMVγ-GFP, respectively. BSMV-α was then linearized with MIuI, BSMV-β was linearized with SpeI, and BSMVγ-ATG8 and BSMVγ-GFP were linearized with BssHII then the linearized vectors were transcribed *in vitro* to produce 5ʹ-capped infectious BSMV RNA molecules using the RiboMAX Large-Scale RNA Production-T7 Kit (Promega, Madison, WI, United States), with a cap analog added to the transcription mixture. They were then mechanically infected with a 1:1:1 mixture of RNAα, RNAβ and RNAγ-ATG6, or RNAγ- GFP in 1 × GKP buffer (50 mM Gly, 30 mM K2HPO3, 1% bentonite and 1% kieselguhr). In the field, inoculation of BSMV was performed at the heading stage by inoculating 50 spikes with 20 µL of BSMV-ATG8 or BSMV-GFP transcript mixture, respectively.

### Immunohistochemistry

Wheat seeds were removed from the spike then treated with 4% paraformaldehyde at 4°C overnight and gradient-dehydrated. The prepared grain tissues were then embedded in paraffin, cut into 7-µm sections, adhered to gelatin-coated glass slides, and dried at 37°C overnight. The slides were then dewaxed, gradient-dehydrated, and digested with 20 µM proteinase K at 37°C for 10 min before blocking in 2% BSA at 37°C for 30 min. Rabbit anti-ATG8 antibody was then added before incubating the slides at 47°C overnight. They were then washed three times with PBS before adding 1 µL secondary antibody (goat anti-rabbit-Alexa Fluor 555 antibody in 10 ml blocking buffer), and incubating at 37°C for 1 h. The nuclei were then stained with 4′, 6-diamidino-2-phenylindole (DAPI) (AnaSpec Inc., San Jose, CA, United States) at room temperature for 10 min. Fluorescence was observed with a fluorescence microscope (HT7700, Hitachi, Tokyo, Japan).

### TUNEL Assay

Wheat seeds were removed from the spike then treated with 4% paraformaldehyde at 4°C overnight. A TUNEL assay was then carried out as described previously (Li et al., 2019).

### Periodic Acid-Schiff Staining

The paraffin-embedded seeds were transected down the middle, placed in xylene for 20 min, anhydrous ethanol for 5 min, and 75% alcohol for 5 min, and then washed with tap water three times for 30 s each time. The sections were then dyed in periodate dye solution for 10 min, rinsed with tap water then distilled water, and dyed in Schaeffer dye solution for 20 min before a final rinse in running water for 5 min. They were then stained in Hematoxylin solution for 3–5 min, rinsed with tap water, and differentiated in 1% HCl alcohol solution for 30 s. Following a final rinse in tap water, they were then placed in blue-back solution for 5 min before rinsing with running water. The prepared slices were examined under a microscope, and images were collected for analysis of cell structure.

## Results

### The Thickness of the Pericarp in the Developing Grains Differs According to the Position on the Spike and the Developmental Stage

**FIGURE 1:**
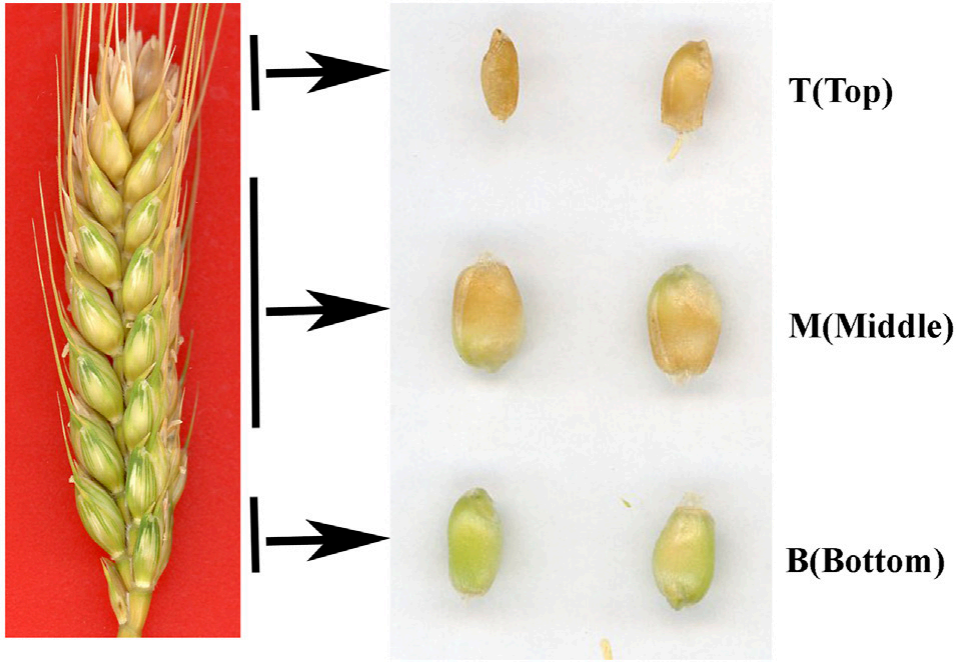
Phenotypes of grains taken from the **top (T),** **middle (M)** and **bottom (B)** of the spike 25 days after flowering.

**FIGURE 2:**
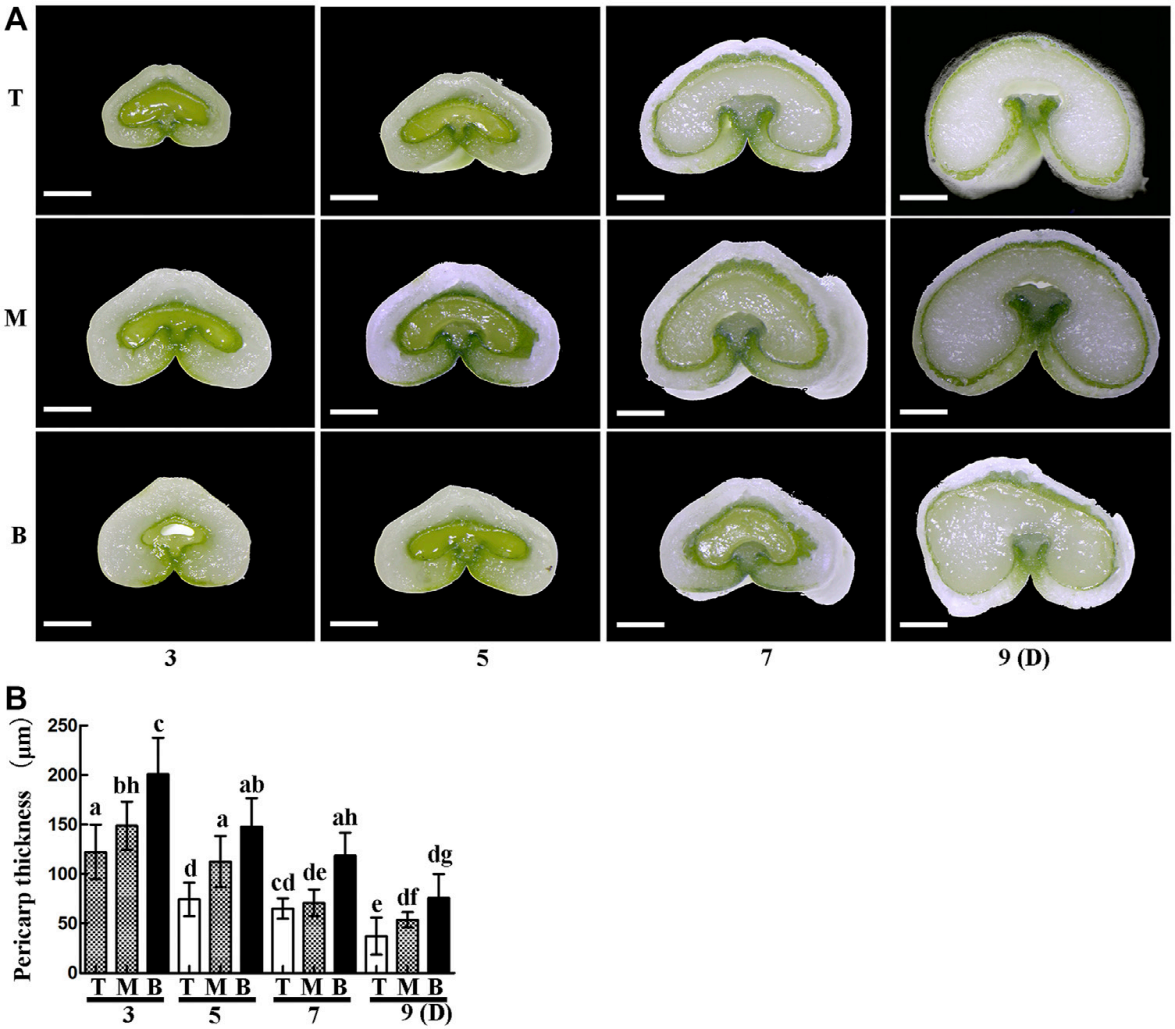
Cross-sections of grains from the **top (T),** **middle (M)** and **bottom (B)** of the spike 3, 5, 7 and 9 days after flowering. **(A)**. Scale bars: 500 µm. **(B)**. Statistical analysis of pericarp thickness at each position on the spike. Bars represent the mean ± SD of three independent experiments. Asterisks indicate a significant difference as determined by ANOVA, and different letters represent a significant difference between positions on the spike.

It is well known that differences in light, temperature and nutrient supply cause wheat grains to mature at different rates in different positions on the spike. In this study, grains at the top of the spike were premature and small, while those in the middle matured faster and were big and full, and those on the bottom matured late and were also relatively small (Figure 1). The thickness of the pericarp was then examined in grains obtained from the top, middle and bottom of the spike at different development stages. Cross-sections revealed that the thickness was smallest at the top of the spike, followed by the middle, then the bottom, with a gradual decrease in thickness with increasing development (Figures 2A,B). These results indicate that pericarp thickness significantly differs at different positions on the spike and at different developmental stages.

### The Loss of Cell Layers Caused by PCD Results in a Decrease in Pericarp Thickness

**FIGURE 3:**
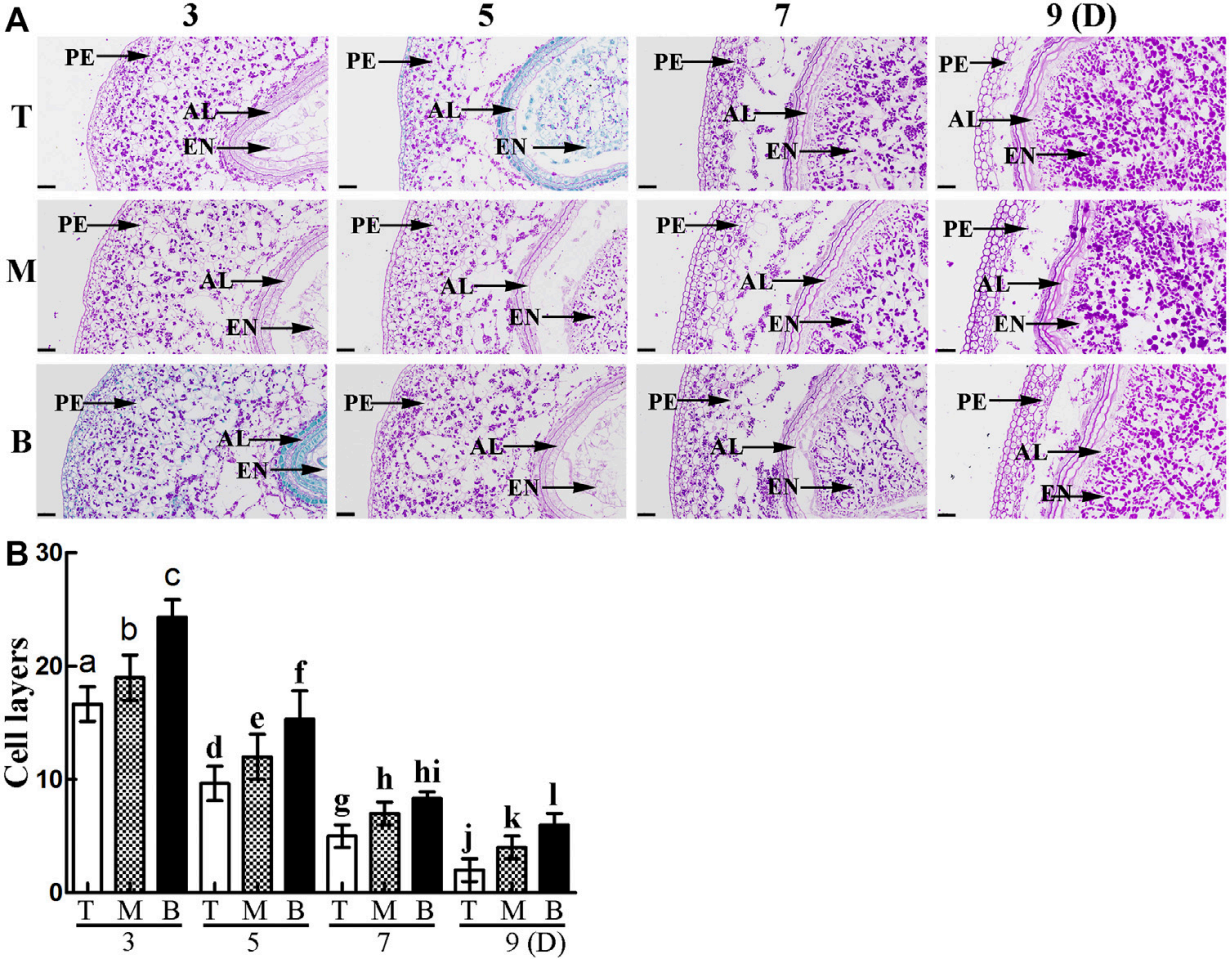
**(A)**. PAS staining showing grain morphology and structure. PE: pericarp, AL: aleurone layer, EN: endosperm. Scale bars: 50 µm. **(B)**. Statistical analysis of cell layers in the pericarp of grains taken from the **top (T),** **middle (M)** and **bottom (B)** of the spike. Bars represent the mean ± SD of three independent experiments. Asterisks indicate a significant difference as determined by ANOVA., and different letters represent a significant difference between positions on the spike.

**FIGURE 4:**
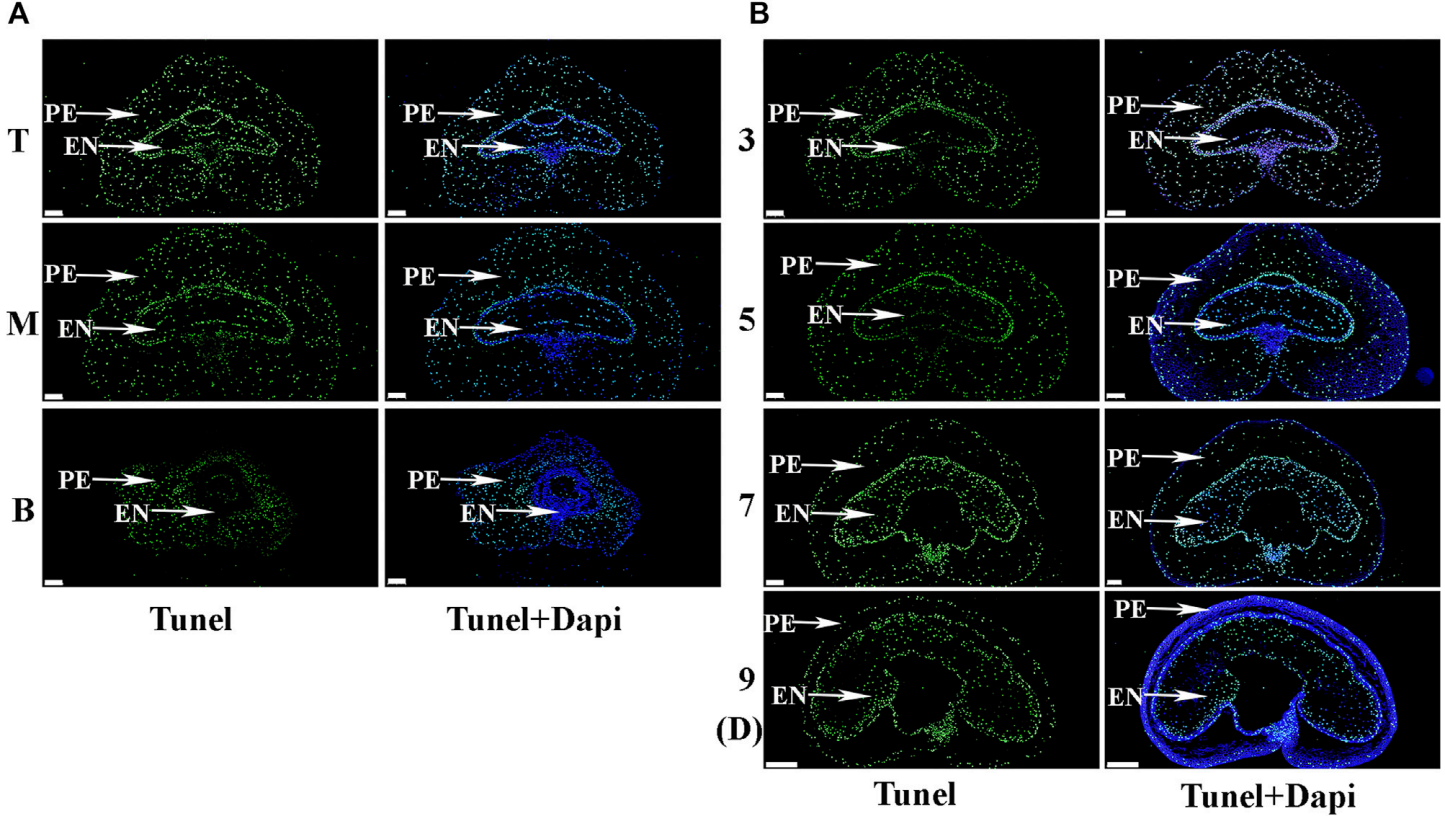
The process of programmed cell death (PCD) in the pericarp during early grain development as detected by TUNEL staining. **(A)**. Images of grains sampled from the **top (T),** **middle (M)** and **bottom (B)** of the spike 3 days after flowering. PE: pericarp, AL: aleurone layer, EN: endosperm. TUNEL images indicate nuclei following PCD, while DAPI images indicate all nuclei. Scale bars: 200 µm. **(B)**. Grains taken from the middle of the spike 3, 5, 7 and 9 days after flowering. Scale bars: 200 µm.

To further explore the differences in pericarp thickness, PAS staining was carried out. The results showed that pericarp samples obtained from the top of the spike had the least number of cell layers, followed by those in the middle, with most numerous layers in those from the bottom (Figure 3A). The number of cell layers also decreased gradually with increasing development (Figure 3B). TUNEL staining is often used to determine PCD in the pericarp (Dominguez et al., 2001), with green fluorescence indicating TUNEL-positive signals in the nuclei indicative of PCD (Zhou et al., 2009). Here, TUNEL signals were extremely intense in pericarp samples from the top of the spike, followed by those in the middle, with weakest signals in those from the bottom, and this trend was consistent for 3 – 9 days after flowering (Figure 4). These data suggest that the reduction in pericarp thickness was the result of a loss in cell layers induced by PCD.

### Autophagy Is Involved in PCD in the Pericarp

**FIGURE 5:**
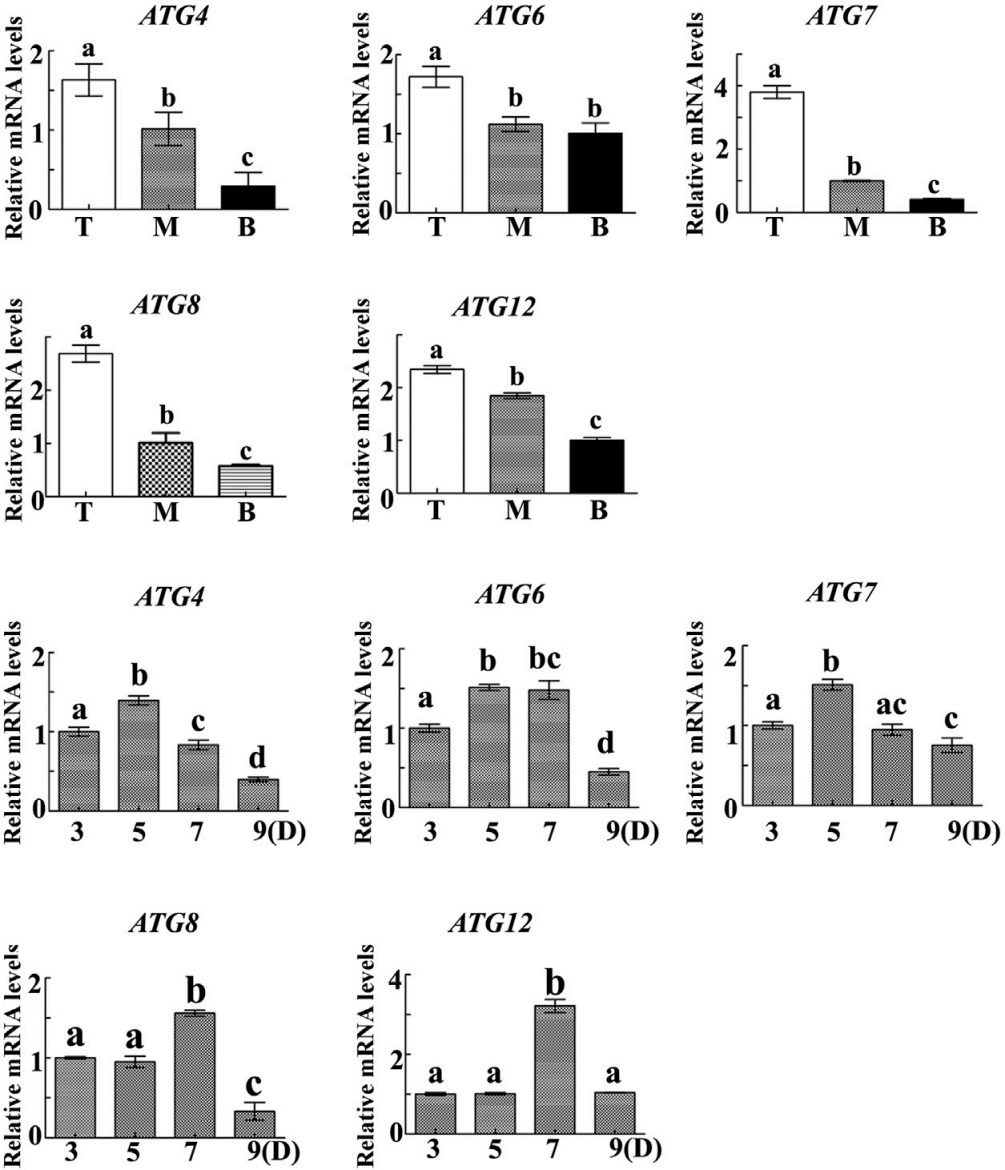
The relative expression of autophagy-related genes in the pericarp of grains taken from the **top (T),** **middle (M)** and **bottom (B)** of the spike 3, 5, 7 and 9 days after flowering. α-Tubulin was used as an internal reference, and the relative expression was calculated using the 2^−ΔΔT^ method. Asterisks indicate significant differences as determined by ANOVA, and different letters represent significant differences between columns.

**FIGURE 6:**
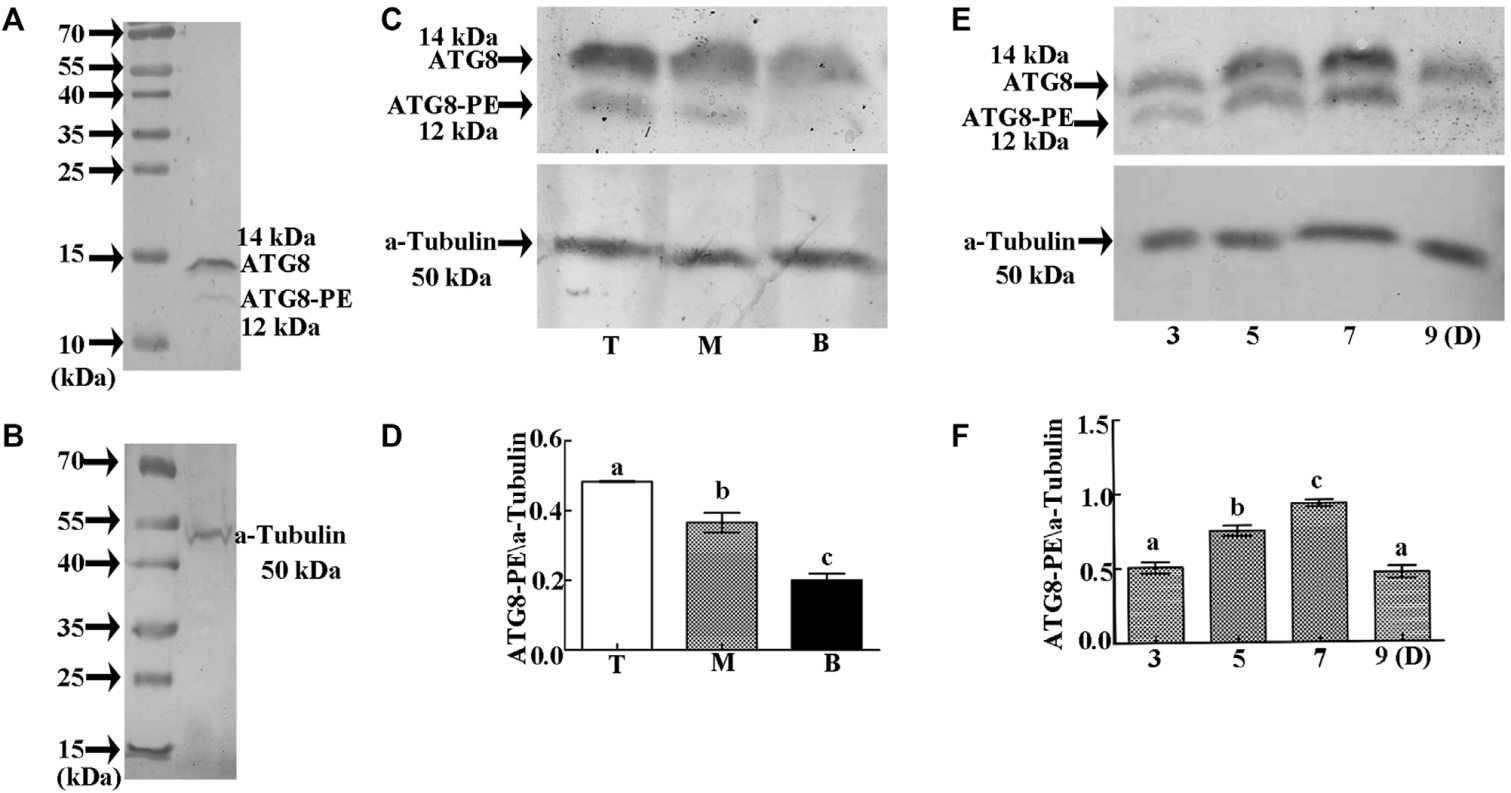
Relative expression of ATG8-PE as detected by Western blotting. **(A)**. Specific detection of ATG8 polyclonal antibody on 15% SDS gel. **(B).** Specific detection of α-tubulin polyclonal antibody on 12.5% SDS gel. **(C).** Relative expression of ATG8-PE in grains taken from the **top (T),** **middle (M),** and **bottom (B)** of the spike 3 days after flowering. **(D).** Statistical analysis of ATG8-PE expression at each position on the spike. **(E).** Relative expression of ATG8-PE at 3, 5, 7 and 9 days **(D)** after flowering. **(F).** Statistical analysis of ATG8-PE expression at each development stage. β-actin was used as a standard. Data represent the mean ± SD of three independent experiments. Asterisks indicate a significant difference as determined by ANOVA. Different letters represent significant differences between columns.

**FIGURE 7:**
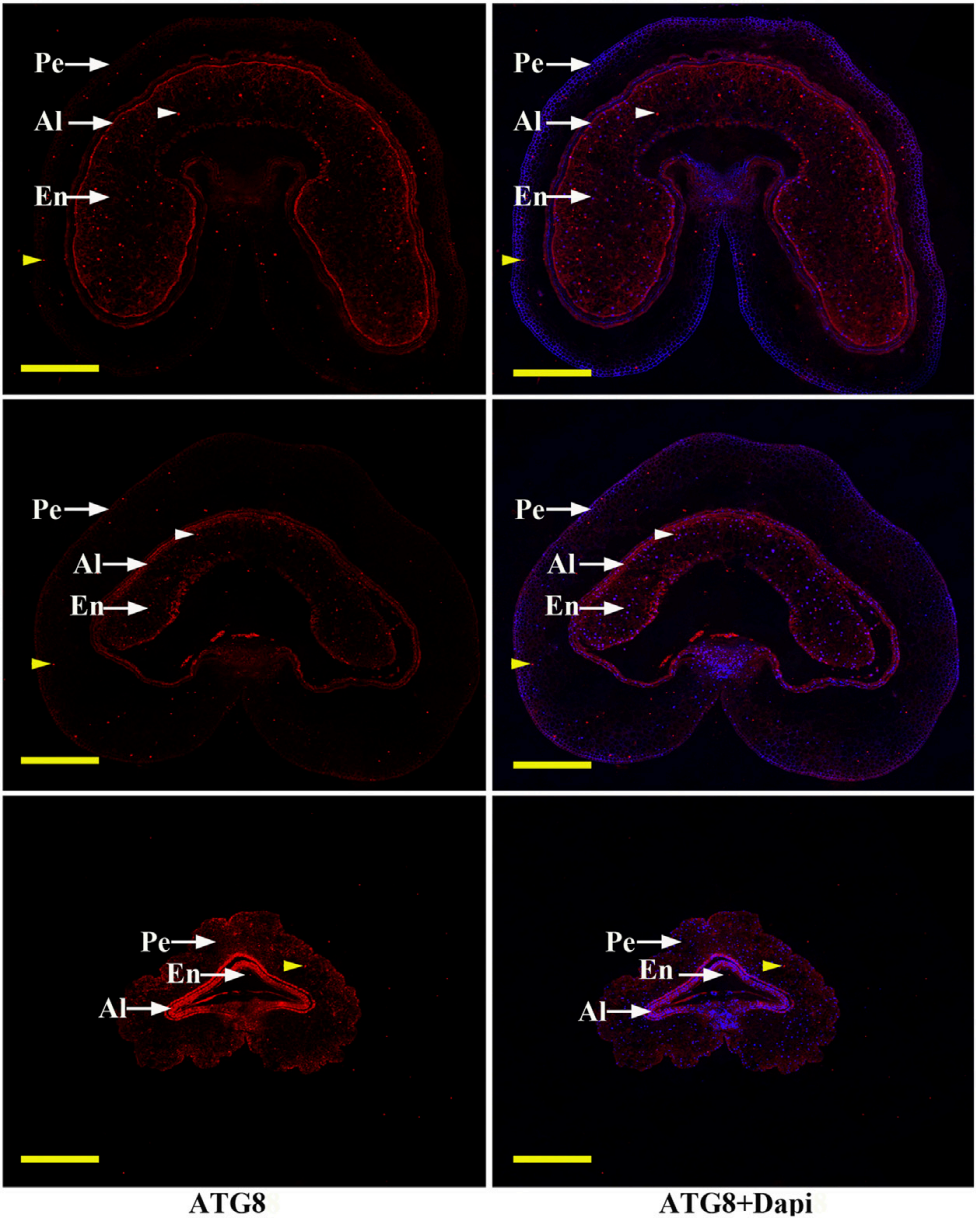
Immunohistochemical analysis of autophagy structures in grains taken from the **top (T),** **middle (M)** and **bottom (B)** of the spike. Short arrows indicate autophagy structures (brown indicate pericarp autophagy structures, white indicate endosperm autophagy structures). Grains were obtained 3 days after flowering, then the pericarp was stained with DAPI and anti-ATG8 antibody followed by Alexa 555-labeled secondary antibody. PE: pericarp, AL: aleurone layer, EN: endosperm; Scale bars: 200 µm.

In order to determine the regulatory mechanism underlying PCD, we examined autophagy in the pericarp of developing grains obtained from different positions of the spike and different development stages. QRT-PCR showed that key autophagy-related genes (*ATG4*, *ATG6*, *ATG7*, *ATG8*, and *ATG12*) were highly expressed in samples from the top of the spike, followed by those in the middle, with weakest expression in those from the bottom (Figure 5). Moreover, highest peaks appeared 5 and 7 days after flowering (Figure 5). Western blotting also showed that the band of ATG8-PE was greatest in the top grains followed by those in the middle, with weakest signal in the bottom grains (Figures 6A–D). Moreover, expression of ATG8-PE was increasing for 3 - 7 days after flowering (Figures 6E,F). In addition, immunohistochemistry showed that most autophagy structures were observed in samples obtained from the top of the spike, followed by those in the middle, with least structures in those from the bottom (Figure 7). These results indicate that autophagy increases from the bottom to the top of the spoke, and is involved in the regulation of PCD in the pericarp during early stages of grain development.

### Inhibition of Autophagy Increases Pericarp Thickness

**FIGURE 8:**
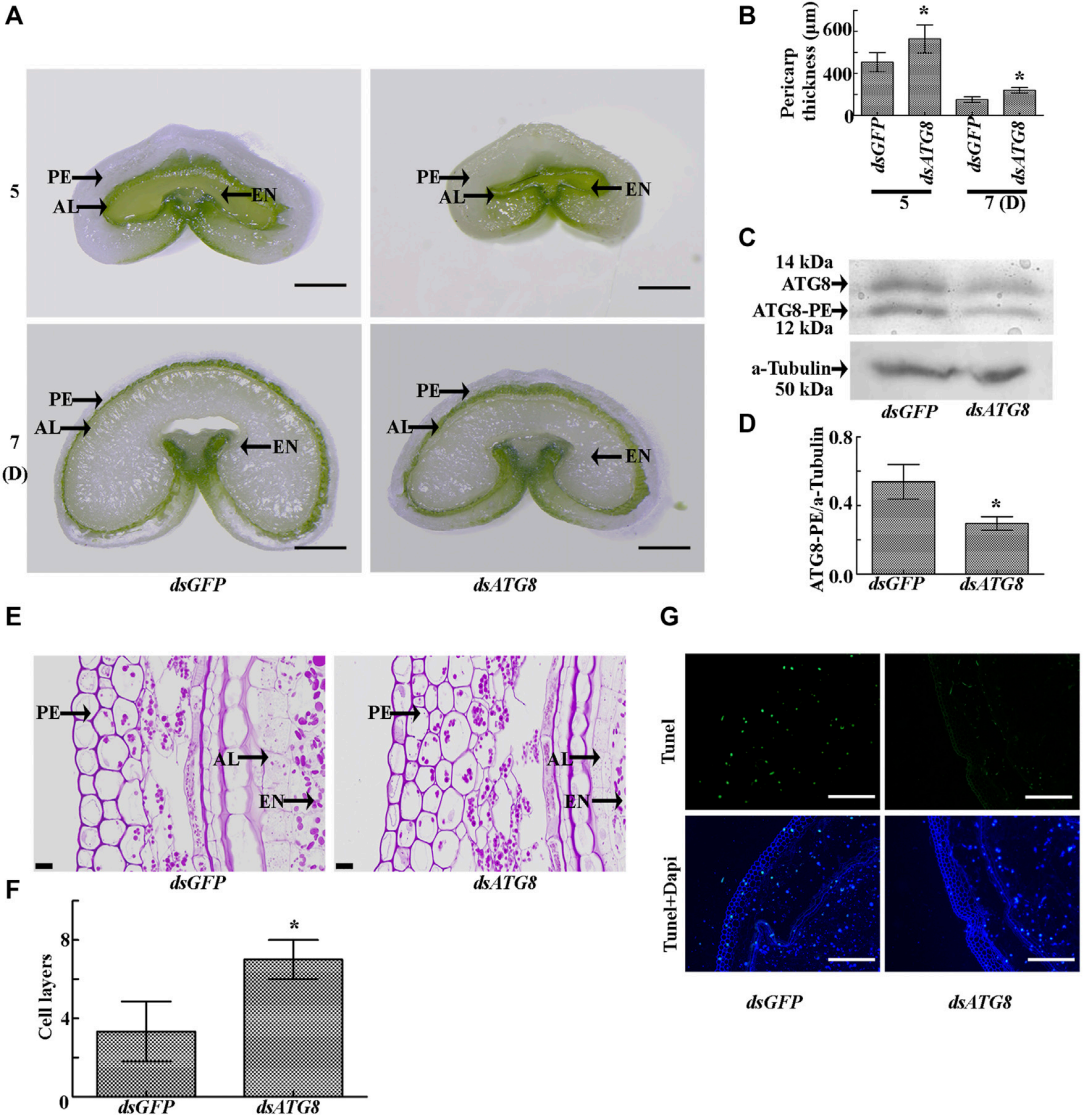
Morphology of the grains after knockdown of *ATG8*. **(A)**. Cross sections of grains sampled five and 7 days after flowering following *ATG8* knockdown. dsGFP was used as a control. PE: pericarp, AL: aleurone layer, EN: endosperm; Scale bars: 500 µm. **(B).** Statistical analysis of pericarp thickness following *ATG8* knockdown. Asterisks indicate a significant difference based on the Student’s t-test, *p* < 0.05. **(C).** Knockdown efficiency of *ATG8* as determined by Western blotting. α-tubulin was used as the internal reference. **(D).** Statistical analysis of the knockdown efficiency of *ATG8*. Asterisks indicate a significant difference based on the Student’s t-test, *p* < 0.05. **(E).** PAS staining 7 days after flowering showing the morphology and structure of the grains following *ATG8* knockdown. Scale bars: 20 μm. **(F).** Statistical analysis of pericarp cell layers following *ATG8* knockdown. Bars represent the mean ± SD of three independent experiments. Asterisks indicate a significant difference according to the Student’s t-test, *p* < 0.05. **(G)**. TUNEL staining 7 days after flowering showing the morphology and structure of the grains following *ATG8* knockdown. Scale bars: 50 μm.

In order to determine the effect of autophagy on PCD, we carried out knockdown of the key autophagy gene (*ATG8*). The results showed that interference of *ATG8* (Figures 8C,D) resulted in a significant increase in the thickness of the pericarp (Figures 8A,B) and the number of cell layers (Figures 8E,F), with an obvious delay in the process of PCD (Figure 8G). These findings indicate that PCD in the pericarp cells of developing wheat grains is dependent on *ATG8*-mediated autophagy.

### Analysis of Wheat Phenotypes After Inhibition of Autophagy

**FIGURE 9:**
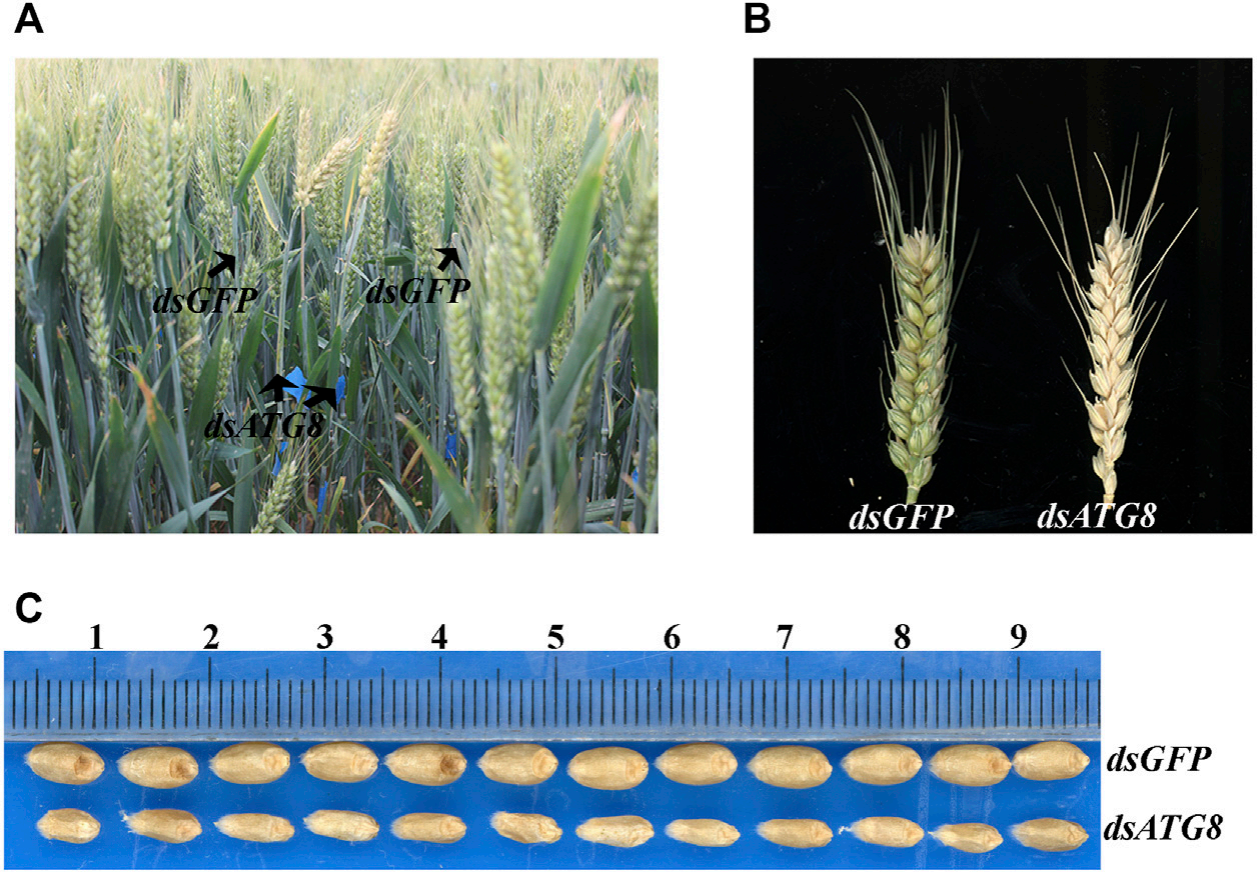
Phenotype of wheat following *ATG8* knockdown. **(A)**. The phenotype of wheat in the field 25 days after flowering following *ATG8* knockdown. **(B)**. The phenotype of the spike 25 days after flowering. **(C)**. mature grains following *ATG8* knockdown.

To further examine the effect of autophagy, changes in the wheat phenotype were also examined following knockdown of *ATG8*. The results showed that interference resulted in earlier maturation by 4 days (Figures 9A,B), with smaller, less full grains (Figure 9C).

## Discussion

PCD is central to the development, homeostasis, and integrity of multi-cellular organisms (Ameisen, 2002). In plant cells, extensive chromatin condensation and degradation of nuclear DNA is one of the most conspicuous features of cells undergoing PCD (Latrasse et al., 2016). For example, during the early development of barley grains, PCD in the pericarp cells provides space and nutrients for subsequent grain filling (Radchuk et al., 2018). Meanwhile, autophagy is responsible for degrading unnecessary components and redistributing nutrients, thereby ensuring normal grain development (Li et al., 2015; Masclaux-Daubresse et al., 2017; Di Berardino et al., 2018). The process of autophagy begins with the formation of a phagophore with a double-membrane cup-shaped structure, which expands to form a double-membrane vesicle called an autophagosome. Upon completion, the autophagosome docks and fuses with the vacuole for cargo degradation (Feng et al., 2014) then the resulting breakdown products are released back into the cytosol to maintain nutrient and energy homeostasis (Yin et al., 2016). However, whether or not autophagy is involved in regulating PCD in the pericarp of developing wheat grains, and the underlying regulatory mechanism, remains unknown. Our data suggest that autophagy is indeed involved, with repression of *ATG8*-mediated autophagy seeming to delay PCD, resulting in an increased thickness and number of cell layers within the pericarp.

High temperatures after anthesis can significantly reduce the grain weight in wheat, thereby causing a reduction in yield (Zahedi et al., 2003; Djanaguiraman et al., 2020). However, grains in different positions of the spike are affected by environmental stress to a differing degree (Yu et al., 2014; Li et al., 2016). Light and temperature stress have the most serious effect on grains at the top of the spike, followed by those in the middle, and lastly, those on the bottom (Steinmeyer et al., 2013). Top grains therefore reach maturity earlier and are smaller in size, while bottom grains mature later, but are also relatively small due to the lack of light and insufficient supply of nutrients. It was previously reported that stress promotes autophagy and PCD in plants (Bassham et al., 2006; Kabbage et al., 2017; Chua et al., 2019). Pericarp and endosperm PCD are necessary for grain maturation (Dominguez and Cejudo, 2014). In this study, both processes occurred most strongly in the pericarp of grains at the top of the spike, followed by those in the middle, and lastly, to a weaker degree, those at the bottom. The top-to-bottom maturation sequence of wheat grain and its peel is actually a process of abiotic stress that causes wheat autophagy and PCD.

Plant development requires specific cells to be eliminated in a predictable and genetically regulated manner referred to as programmed cell death (PCD). However, the target cells do not merely die but they also undergo autophagy to degrade their cellular corpses (Escamez and Tuominen, 2017). In plants, controversy remains over the regulatory effect of autophagy on PCD. One viewpoint is that autophagy negatively regulates PCD; for example, mutation of autophagy was found to result in leaf senescence in Arabidopsis (Hanaoka et al., 2002), and autophagy in plants was found to eliminate reactive oxygen species induced by various abiotic stresses (Avin-Wittenberg, 2019). Meanwhile, the opposing view suggests that autophagy promotes PCD; for example, mutation of autophagy caused degradation of rice pollen tapetum (Kurusu et al., 2014), and autophagic components contributed to hypersensitive cell death in Arabidopsis (Hofius et al., 2009). In this study, however, the characteristics of autophagy and PCD coexisted in the pericarp during early development of the wheat grains, providing an ideal model for studies on the relationship between autophagy and PCD.

Autophagy occurs not only in the pericarp, but also in the embryo and endosperm of grain (Steinmeyer et al., 2013). At the whole-plant level, autophagy is an essential process for nutrient remobilization from leaves to seeds, and is fundamental for seed filling (Avin-Wittenberg, 2019). In *ATG8*-RNAi lines of rice, autophagic activity was slightly inhibited, grain yield and quality were reduced, and grains matured early (Fan et al., 2020). In our study, knockdown of wheat *ATG8* also resulted in early maturity and smaller grains. The main reason is that inhibition of *ATG8*-mediated autophagy leads to the decrease of nutrient recycling into wheat grains. So, it can be inferred that other spike positions will also show the corresponding phenomena of smaller grains and early maturity after *ATG8* interference.

The *ATG8* gene is an evolutionarily conserved gene that is expressed in various plant tissues (Boycheva Woltering and Isono, 2020). In rice, *ATG8* interference lines exhibited abnormal roots, a reduced number of grains per panicle, and other unfavorable agronomic traits (Fan et al., 2020). It is therefore difficult to rule out the negative effects of *ATG8* knockout on other agronomic traits in analysis of grain phenotypes. In this study, we therefore carried our BSMV-mediated transient interference of *ATG8* to minimize interference of other agronomic traits. As a result, PCD was weakened, and the thickness of the pericarp increased, suggesting strong dependency on ATG8-mediated autophagy, and a positive regulatory effect. Overall, the findings suggest that positive and negative regulation of PCD by autophagy mainly depends on the types of plant tissue and stress. Autophagy is dependent on a set of autophagy-related (ATG) proteins, of which the ubiquitin-like protein ATG8 plays a central role, functioning in autophagosome formation, and mediating membrane tethering, elongation and fusion (Nakatogawa et al., 2007). Upon autophagy activation, ATG8 undergoes lipidation to generate a membrane-bound ATG8-phosphatidylethanolamine (ATG8-PE) conjugate that localizes on growing phagophores and autophagosomes. ATG8 proteins are therefore often used as reliable markers to assess the induction and progression of autophagy (Yoshimoto et al., 2004). In this study, ATG8 was required for PCD in the pericarp of developing wheat grains. ATG8 lipidation occurs in both the outer and inner membrane of the phagophore, the precursor to autophagosomes, and is involved in autophagosome formation, as well as the recognition of specific cargo specifically targeted for autophagy (Kellner et al., 2017). These cargo receptors then interact with ATG8 proteins *via* short peptide motifs known as AIMs (ATG8-family interacting motifs) (Fracchiolla et al., 2017). Vacuolar processing enzymes (VPEs), a class of conserved cysteine proteases, are also involved in plant PCD (Hatsugai et al., 2004; Rojo et al., 2004). For example, VPE4 in barley is required for PCD in the pericarp during grain development (Radchuk et al., 2018), while VPE1 in tomato is translocated to the vacuole through the autophagy pathway, co-localizing with ATG8 in the autophagosomes and autolysosomes to induce PCD (Teper-Bamnolker et al., 2020). However, whether VPE1 interacts with ATG8, and is transported to the vacuole to exert its function on PCD requires further clarification.

## Conclusion

The findings of this study suggest that autophagy and PCD coexist in the pericarp during the early development of wheat grains. Moreover, autophagy and PCD increased from the bottom to the top of the spike, and PCD was dependent on *ATG8*-mediated autophagy. Meanwhile, following knockdown of *ATG8*, the thickness of the pericarp increased, resulting in small premature grains. Overall, this dependence between autophagy and PCD determines the early development of grain pericarp.

## Data Availability

The datasets presented in this study can be found in online repositories. The names of the repository/repositories and accession number(s) can be found below: https://www.ncbi.nlm.nih.gov/genbank/, KF294798.1 https://www.ncbi.nlm.nih.gov/genbank/, AM075827.1 https://www.ncbi.nlm.nih.gov/genbank/, KF294804.1 https://www.ncbi.nlm.nih.gov/genbank/, FJ750848.1 https://www.ncbi.nlm.nih.gov/genbank/, KF294819.1 https://www.ncbi.nlm.nih.gov/genbank/, U76558.1.
